# A Negatively Charged Residue Stabilizes the Tropoelastin N-terminal Region for Elastic Fiber Assembly*

**DOI:** 10.1074/jbc.M114.606772

**Published:** 2014-10-23

**Authors:** Giselle C. Yeo, Clair Baldock, Steven G. Wise, Anthony S. Weiss

**Affiliations:** From the ‡ School of Molecular Bioscience and; § Charles Perkins Centre, University of Sydney, Sydney, New South Wales 2006, Australia,; the ¶ Wellcome Trust Centre for Cell-Matrix Research, Faculty of Life Sciences, University of Manchester, Manchester M13 9PT, United Kingdom,; the ‖ Heart Research Institute, Sydney, New South Wales 2042, Australia, and; the ** Sydney Medical School and; ‡‡ Bosch Institute, University of Sydney, Sydney, New South Wales 2006, Australia

**Keywords:** Aspartate (Aspartic Acid), Elastin, Protein Self-assembly, Small Angle X-ray Scattering, Tertiary Structure, Domain 6, Elastic Fiber Assembly, N-terminal Region, Tropoelastin

## Abstract

Tropoelastin is an extracellular matrix protein that assembles into elastic fibers that provide elasticity and strength to vertebrate tissues. Although the contributions of specific tropoelastin regions during each stage of elastogenesis are still not fully understood, studies predominantly recognize the central hinge/bridge and C-terminal foot as the major participants in tropoelastin assembly, with a number of interactions mediated by the abundant positively charged residues within these regions. However, much less is known about the importance of the rarely occurring negatively charged residues and the N-terminal coil region in tropoelastin assembly. The sole negatively charged residue in the first half of human tropoelastin is aspartate 72. In contrast, the same region comprises 17 positively charged residues. We mutated this aspartate residue to alanine and assessed the elastogenic capacity of this novel construct. We found that D72A tropoelastin has a decreased propensity for initial self-association, and it cross-links aberrantly into denser, less porous hydrogels with reduced swelling properties. Although the mutant can bind cells normally, it does not form elastic fibers with human dermal fibroblasts and forms fewer atypical fibers with human retinal pigmented epithelial cells. This impaired functionality is associated with conformational changes in the N-terminal region. Our results strongly point to the role of the Asp-72 site in stabilizing the N-terminal segment of human tropoelastin and the importance of this region in facilitating elastic fiber assembly.

## Teaser

**Background:** Negative residues are rare in human tropoelastin, and their contributions to protein structure and function are unknown.

**Results:** Mutating aspartate 72 reduces coacervation, cross-linking, and elastogenesis and alters the N-terminal conformation.

**Conclusion:** Aspartate 72 residue stabilizes the N-terminal structure for functional assembly into elastic fibers.

**Significance:** This work provides the first direct evidence of the importance of the tropoelastin N-terminal structure in elastic fiber assembly.

## Introduction

Elastic fibers within the extracellular matrix provide structural integrity and resilience for the mechanical stretching of elastic tissues such as skin, lungs, and vasculature. The main component of elastic fibers is elastin, which is assembled from the tropoelastin monomer.

The contributions of specific tropoelastin regions to elastic fiber assembly have not yet been fully defined. Previous studies have predominantly implicated the tropoelastin central hinge, bridge, and C-terminal foot regions as essential to the coacervation, microfibrillar deposition, and cross-linking stages of elastogenesis (1–5). However, the role of the preceding N-terminal coil region in elastin assembly is less well understood. This region likely contributes to the overall structure of the tropoelastin coacervate, as suggested by the conformational transitions displayed by an isolated domain 2-7 peptide upon an increase in solvent hydrophobicity (6). The N-terminal segment may also facilitate tropoelastin integration into the microfibrillar scaffold via interactions with fibrillin-1 (7). In addition, there are at least three intramolecular cross-links that occur in the region spanning domains 6–15, as well as a number of intermolecular cross-links with the central or C-terminal regions of another molecule (8, 9). A major cross-linking site in elastin is formed through the association of domains 10, 19, and 25, in which the domain 10 sequence of one tropoelastin strand bridges the desmosine-linked domains 19 and 25 of a second molecule via two lysinonorleucine links (10). The likely importance of the tropoelastin N-terminal region in elastogenesis is further supported by the association of a domain 12 mutation with a cutis laxa phenotype that is marked by deficient dermal elastic fibers (11).

Many of the functional and structural properties of tropoelastin are modulated by the abundant positively charged residues within the molecule. For example, tropoelastin binding to fibroblasts is mediated by the basic C-terminal RKRK cluster (12, 13). Tropoelastin deposition onto microfibrils is proposed to be facilitated by electrostatic interactions with microfibril-associated glycoproteins (14, 15). Cross-linking reactions that stabilize the nascent elastic fiber directly involve the positively charged lysine residues within tropoelastin (16). Positively charged residues may also be involved in maintaining the tertiary structure of the protein, as demonstrated by the conformational changes exhibited by an arginine-to-alanine tropoelastin mutant (1).

In contrast to the more recognized roles of the positively charged residues in tropoelastin function, little has been described of the rarely occurring negatively charged residues. One such residue, Asp-72, resides within cross-linking domain 6 of the tropoelastin N-terminal region. Inspection of mammalian tropoelastin sequences (17) indicated that the Asp-72 site is conserved in human, chimpanzee, baboon, and pig tropoelastin. In the cat and cow tropoelastin sequences that do not possess a corresponding Asp-72, a glutamate exists within the same domain upstream of this site.

Our paper investigates the functional significance of a conserved negatively charged residue within the tropoelastin N-terminal region. We produced a human tropoelastin construct with an alanine substitution at the Asp-72 site (D72A) and analyzed its functionality with respect to coacervation, cross-linking, cell interaction, and elastic fiber assembly in comparison with the wild-type (WT) isoform. We also compared the solution structures of the WT and D72A constructs to determine differences in monomer conformation that can account for differences in assembly properties. Our results point to the role of the Asp-72 site in stabilizing the N-terminal segment of human tropoelastin, and the importance of this under-characterized region in facilitating fiber assembly.

## EXPERIMENTAL PROCEDURES

### 

#### 

##### Tropoelastin Production

**FIGURE 1.**
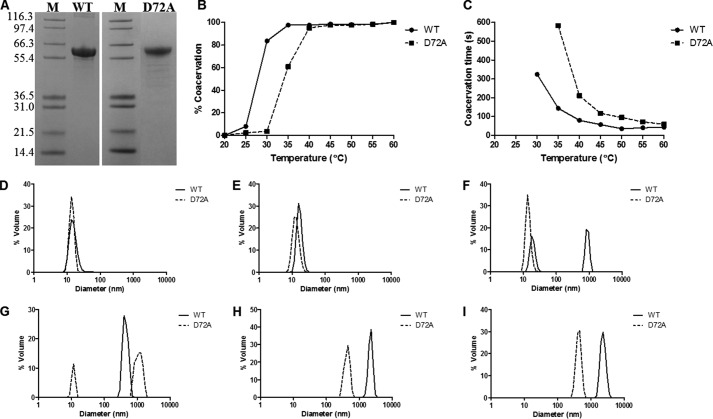
*A,* SDS-PAGE of purified WT and D72A tropoelastin with standard protein markers (*M*) in kDa. *B–I,* coacervation profiles of WT and D72A tropoelastin. *B,* extent of coacervation at each temperature as indicated by relative sample turbidity. *C,* time required to achieve maximum coacervation at each temperature. *D–I,* particle sizes of WT and D72A tropoelastin solutions at 20 °C (*D*), 25 °C (*E*), 30 °C (*F*), 35 °C (*G*), 40 °C (*H*), and 45 °C (*I*).

Bacterial plasmid containing the WT tropoelastin sequence (recombinant wild-type human tropoelastin without domain 26A, corresponding to residues 27–724 of GenBank^TM^ entry AAC98394) was modified by site-directed mutagenesis (GenScript) to obtain the D72A isoform. D72A tropoelastin was purified from transformed *Escherichia coli* BL21 culture, as described previously (18), and verified by plasmid sequencing, SDS-PAGE (Fig. 1*A*), and comparative MALDI-TOF mass spectrometry. WT tropoelastin was produced in-house via a similar, large scale *E. coli* expression system.

Human tropoelastin has been reported to contain low, variable levels of hydroxyproline, ranging from 0 (19) to <30% of all proline residues (20–22). To date, proline hydroxylation has no known role in tropoelastin function, and it has been hypothesized to be a coincidental by-product of collagen hydroxylation (23). This is consistent with the fact that the potential proline hydroxylation sites are not modified in every tropoelastin molecule (20). The absence of hydroxyprolines does not affect tropoelastin secretion, cross-linking, or incorporation into the elastic matrix (21, 24). However, proline over-hydroxylation in tropoelastin has been associated with structural changes, impaired coacervation, and reduced cross-linking and pathological elastin formation (21, 25). For these reasons, although the recombinant WT tropoelastin used in this study is not post-translationally modified, it is expected to be equivalent to the native, mature form of human tropoelastin (26).

##### Coacervation

Light scattering of 10 mg/ml WT and D72A tropoelastin in 0.01 m phosphate-buffered saline (PBS) was monitored by measuring absorbance at 300 nm at 20–60 °C over 10 min in a Shimadzu UV-1601 spectrophotometer. The samples were cooled at 4 °C for 10 min between each temperature change.

##### Particle Size Analysis

Particle sizes of 10 mg/ml WT and D72A tropoelastin solutions were determined via dynamic light scattering using a Malvern Zetasizer Nano (Malvern Instruments). The samples were equilibrated for 5 min at each temperature. At least 40 measurements were taken per sample and averaged to obtain the relative volume percentages of solution particle sizes.

##### Hydrogel Construction

WT and D72A tropoelastin (100 mg/ml in PBS) were cross-linked with 10 mm bis(sulfosuccinimidyl) suberate at 37 °C for 16 h to form hydrogels.

##### Micro-computed Tomography

Lyophilized hydrogels were scanned with a SkyScan 1072 micro-computed tomography system using a 60-kV x-ray beam at a resolution of 3.23 μm. The x-ray projection images were converted into a stack of cross-sections with the NRecon 1.4.4 cone-beam reconstruction program (SkyScan) and rendered into a three-dimensional structure with VGStudio Max 1.2.1 (Volume Graphics GmbH). Hydrogel porosity was calculated using the CTan software (SkyScan).

##### Scanning Electron Microscopy

Hydrogels were mounted with carbon glue, sputter-coated with a 25-nm gold layer, and imaged onto a Zeiss EVO-50 scanning electron microscope.

##### Hydrogel Swelling

Lyophilized hydrogels of known mass were submerged in Milli-Q water (Millipore) for 24 h at 4, 25, and 37 °C. Excess water was drained, and the hydrogels were weighed to calculate water absorption per gram of protein.

##### Cell Attachment

Tissue culture plastic wells were coated with increasing concentrations of WT or D72A tropoelastin at 4 °C overnight and then washed with PBS to remove unbound tropoelastin. Wells were blocked for 1 h with 10 mg/ml heat-denatured bovine serum albumin in PBS. Human dermal fibroblasts (GM3348) grown in Dulbecco's modified Eagle's medium (DMEM) with 10% (v/v) fetal bovine serum were trypsinized and resuspended in serum-free DMEM. Wells were seeded at a density of 1.5 × 10^5^ cells/cm^2^. Standards with known seeding density were added to uncoated and unblocked wells. Cells were allowed to attach at 37 °C for 1 h. Nonadherent cells were washed off with PBS, and the adhered cells were fixed with 3% (w/v) formaldehyde for 20 min and stained with 0.1% (w/v) crystal violet in 0.2 m MES, pH 5.0, for 1 h. Excess stain was washed off with water, and the crystal violet was solubilized with 10% (w/v) acetic acid. Absorbance was read at 570 nm. Standard values were fitted to a linear regression and used to convert sample absorbance values into percentage cell attachment.

##### Antibody Detection (ELISA)

Wells were coated with increasing concentrations of tropoelastin at 4 °C overnight and washed with PBS to remove unbound tropoelastin. Wells were blocked with 3% (w/v) BSA for 1 h. Bound tropoelastin was separately detected with one of three primary antibodies as follows: (*a*) 1:2000 BA4 mouse anti-elastin antibody (Sigma); (*b*) 1:500 rabbit anti-C-terminal antibody (custom-made by Biomatik); or (*c*) 1:5000 mouse anti-domain 6 antibody (custom-made by AbMart). Wells were washed and incubated with 1:5000 goat anti-mouse or anti-rabbit IgG conjugated with horseradish peroxidase for 1 h. Wells were visualized with ABTS solution (1.04 mg/ml 2,2′-azino-bis(3-ethylbenzothiazoline-6-sulfonic acid, 0.05% (v/v) H_2_O_2_, 10 mm CH_3_COONa, 5 mm Na_2_HPO_4_) at 37 °C for 1 h, and absorbance readings were measured at 405 nm.

##### Immunostaining of Elastic Fibers

Human dermal fibroblasts (GM3348; obtained from the Coriell Research Institute) cultured in DMEM with 10% (v/v) fetal bovine serum and 1% (v/v) penicillin/streptomycin and human retinal pigmented epithelium cells (ARPE-19; obtained from Dr. M. Madigan, Save Sight Institute, New South Wales, Australia) cultured in DMEM/Nutrient mixture F-12 with 10% (v/v) fetal bovine serum, 2 mm l-glutamine, and 1% (v/v) penicillin/streptomycin were seeded on glass coverslips at 18,400 cells/cm^2^. At 10 and 14 days post-seeding, respectively, 20 μg/ml WT or D72A tropoelastin was added to the GM3348 and ARPE-19 cultures. At 1, 4, 7, and 10 days after tropoelastin addition, cells were fixed with 4% (w/v) paraformaldehyde for 20 min and quenched with 0.2 m glycine. The cells were incubated with 0.2% (v/v) Triton X-100 for 6 min, blocked with 5% (w/v) bovine serum albumin at 4 °C overnight, and stained with 1:500 BA4 mouse anti-elastin antibody for 1.5 h and 1:100 anti-mouse IgG-FITC antibody (Sigma) for 1 h. The coverslips were mounted onto glass slides with ProLong Gold anti-fade reagent with DAPI (Invitrogen).

##### Confocal Microscopy

Immunostained samples were visualized with an Olympus FluoView FV1000 confocal microscope using laser excitation at 488 nm to detect FITC fluorescence and 559 nm to detect elastin autofluorescence. Z-stacks were taken from areas distributed across each sample and converted to maximum projection images. Confocal images of WT and D72A elastic fibers were analyzed using ImageJ (rsbweb.nih.gov).

To compare fiber fluorescence or autofluorescence, a threshold was set to exclude background and saturated pixel intensities. The average intensity of pixels within this threshold was measured for each projection image and averaged for each sample. To compare fiber abundance, two perpendicular reference lines were drawn in each projection image. The number of fibers intersecting either reference line was counted and averaged for each sample. Fiber width was measured and averaged from a total of ∼150 randomly selected sections of WT or D72A elastic fibers. The area occupied by cell nuclei was used as a control of cell number and viability in all samples.

##### Circular Dichroism (CD)

Far-UV CD spectra of 0.15 mg/ml WT and D72A tropoelastin in 10 mm phosphate and 150 mm NaF were recorded on a Jasco J-815 spectrometer equipped with a Peltier-controlled sample chamber. Samples were scanned with a bandwidth of 1.0 nm at 20 nm/min. Each spectrum was averaged from five scans, buffer-corrected, and smoothed using 3-point adjacent averaging. Secondary structure composition was estimated from the CD spectrum using the CONTINLL and CDSSTR methods (27) with a reference set of 37 soluble proteins.

##### Small Angle X-ray Scattering

WT and D72A tropoelastin were dissolved in PBS to 20 and 16 mg/ml, respectively, and mixed with 2 mm dithiothreitol. Small angle x-ray scattering data were collected at the ESRF on BM29 with a detector distance of 2.8 m using the BioSAXS robot. The scattering images obtained were spherically averaged using in-house software and buffer scattering intensities subtracted using PRIMUS (28). Particle shapes were generated *ab initio* using GASBOR (29). Multiple GASBOR runs were performed to generate 10 similar shapes that were combined and filtered to produce an averaged model using the DAMAVER software package (30).

##### Statistical Analyses

Replicate values were reported as means ± S.E. Statistical significance was calculated using two-way analysis of variance. Significance was set at *p* < 0.05 or higher.

## RESULTS

### 

#### 

##### D72A Tropoelastin Shows Decreased Propensity for Self-association

WT and D72A tropoelastin solutions were analyzed spectrophotometrically at different temperatures to determine the extent of protein self-association. WT coacervated fully at 35 °C, although D72A required a higher temperature of 40 °C to attain full coacervation (Fig. 1*B*). At temperatures up to 50 °C, D72A also consistently coacervated more slowly than WT, although the difference was most pronounced within the physiological temperature range (Fig. 1*C*).

To confirm differences in the coacervation behavior of WT and D72A, the particle sizes of the tropoelastin solutions were assessed via dynamic light scattering over a temperature range (Fig. 1, *D–I*). At 20 and 25 °C, both WT and D72A species were in the 10–15-nm monomer form. At 30 °C, ∼43% of WT molecules self-associated, whereas the D72A species remained as monomers. At 35 °C, all of the WT but only ∼76% of D72A were in the coacervate form. At 40 °C, all D72A molecules coacervated fully, although the diameter of these mutant assemblies at ∼0.5 μm was significantly smaller than that of the WT at ∼2.3 μm. This difference between the WT and D72A coacervate sizes was observed even at higher temperatures, up to 45 °C.

##### D72A Tropoelastin Cross-links into Structurally Different Hydrogels

**FIGURE 2.**
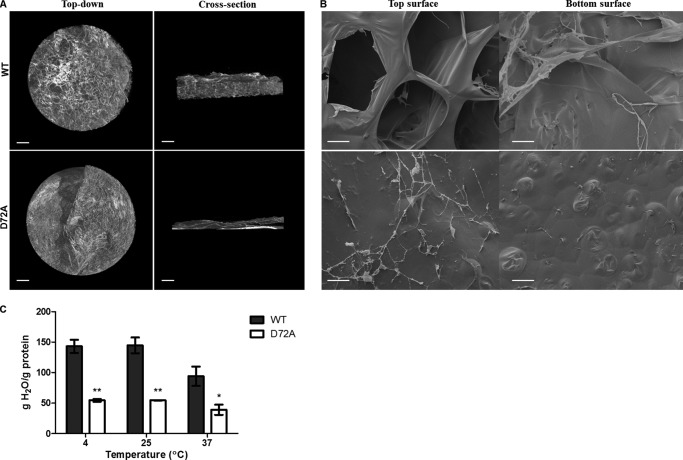
**Morphological and swelling properties of hydrogels.** *A,* micro-computed tomography images showing the top-down and cross-section views of WT and D72A hydrogels. *Scale bar,* 0.5 mm. *B,* scanning electron microscopy images of the top and bottom surfaces of WT and D72A hydrogels. *Scale bar,* 100 μm. *C,* swelling of WT and D72A hydrogels in water at 4, 25, and 37 °C. The amount of water absorbed is expressed as a ratio to hydrogel mass. *, *p* < 0.05; **, *p* < 0.005.

WT and D72A tropoelastin incubated with 10 mm bis(sulfosuccinimidyl) suberate for 16 h polymerized completely to form hydrogels, as evidenced by the absence of tropoelastin monomers in the aqueous solution left after hydrogel formation (data not shown). Micro-computed tomography reconstruction of the WT and D72A hydrogels indicated distinct structural differences between the constructs (Fig. 2*A*). The D72A hydrogel was composed of discrete thin layers of densely stacked material. This morphology contrasted greatly with the open fibrous network that spanned the full thickness of the WT hydrogel. Analysis of the projection slices across the *z* axis of the hydrogels confirmed that the WT material had a significantly higher porosity at 90.2 ± 0.6% than the D72A material at 59.4 ± 1.3%.

Scanning electron microscopy imaging of the hydrogels at higher resolution revealed surface morphology differences between the WT and D72A constructs (Fig. 2*B*). Although the top surface of the WT hydrogel showed a highly porous, honeycomb-like network with open channels ∼150–200 μm in diameter, the top surface of the D72A hydrogel comprised a solid sheet with fewer and smaller pores, averaging 20–40 μm. Thin fibers were present on the D72A hydrogel surface and appeared to be in the process of coalescing into the surface layer. The top surface of the D72A hydrogel reflected a similar morphology to the bottom surfaces of both WT and D72A cross-linked constructs, which were characterized by a smooth dense sheet with infrequent, smaller pores of ∼20 μm.

The WT and D72A hydrogels also exhibited differences in swelling behavior (Fig. 2*C*). When submerged in water for 24 h, the D72A hydrogels expanded less (4.7 ± 0.04-fold increase) than the WT constructs (6.4 ± 0.4-fold increase) compared with the pre-swollen samples. Consistent with this smaller volume increase, the amount of water absorbed by D72A hydrogels was 58–62% less than that of WT hydrogels. This significant decrease in swelling by mutant hydrogels was observed consistently at 4, 25, and 37 °C.

##### D72A Tropoelastin Binds Human Dermal Fibroblasts

**FIGURE 3.**
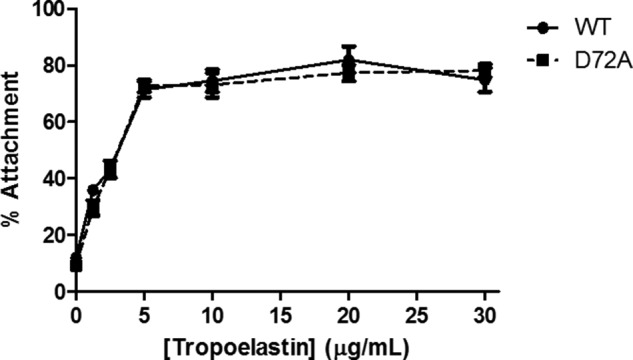
**Attachment of human dermal fibroblasts to tissue culture wells coated with WT and D72A tropoelastin.**

WT and D72A tropoelastin exhibited no significant difference in their ability to support the attachment of human dermal fibroblasts (Fig. 3). Cell binding to WT- or D72A-coated tissue culture plastic increased proportionally with tropoelastin concentration until a plateau at ∼5 μg/ml tropoelastin. At saturation levels of tropoelastin coating, ∼75% of seeded cells adhered to either WT or D72A.

##### D72A Tropoelastin Shows Impaired Elastic Fiber Assembly

**FIGURE 4.**
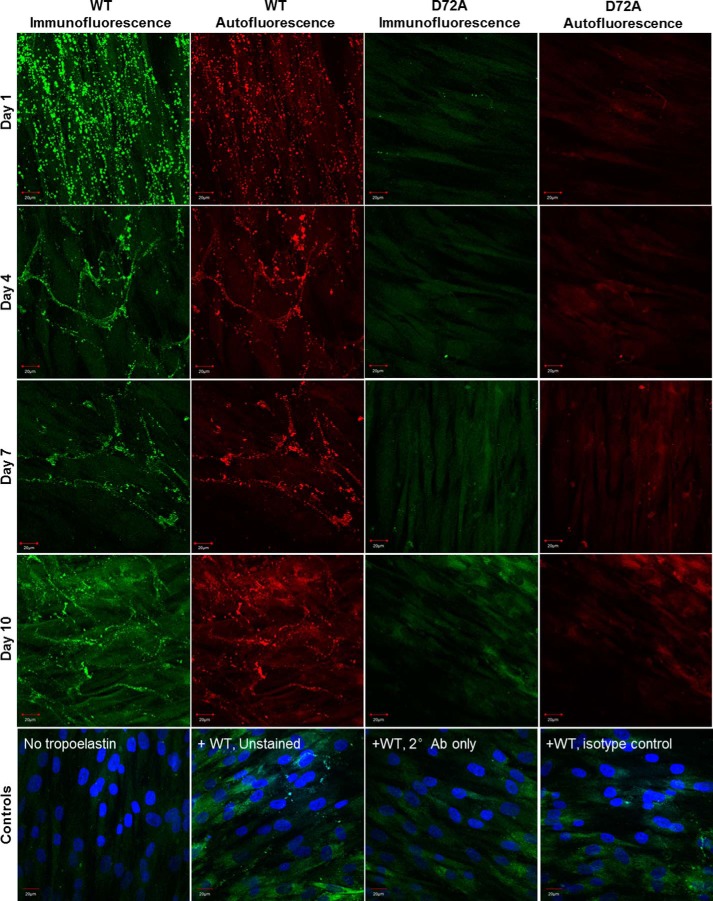
**Confocal microscope images of elastic fibers formed from exogenous WT and D72A tropoelastin in human dermal fibroblast cultures, at 1, 4, 7, and 10 days after tropoelastin addition.** Immunofluorescence and autofluorescence signals from each representative field of view are shown. Staining controls include cells with no added tropoelastin and samples with added tropoelastin but are unstained, have no primary antibody, or have a nonspecific primary mouse antibody with the anti-mouse secondary antibody. Staining controls show immunofluorescence signals as well as DAPI-stained cell nuclei. *Scale bar,* 20 μm.

WT and D72A tropoelastin showed markedly different capacities for elastic fiber formation when added to the culture medium of GM3348 human dermal fibroblasts (Fig. 4). One day after addition into the extracellular environment, WT globules were clustered linearly as a precursor step to fiber development. In contrast, D72A particles assembled into a few visible spherules, which were dispersed throughout the extracellular matrix. Four days after tropoelastin addition, a branched network of WT elastic fibers was visible, but no D72A spherules or fibers were present. The same trend persisted for 7 and 10 days after tropoelastin addition.

**FIGURE 5.**
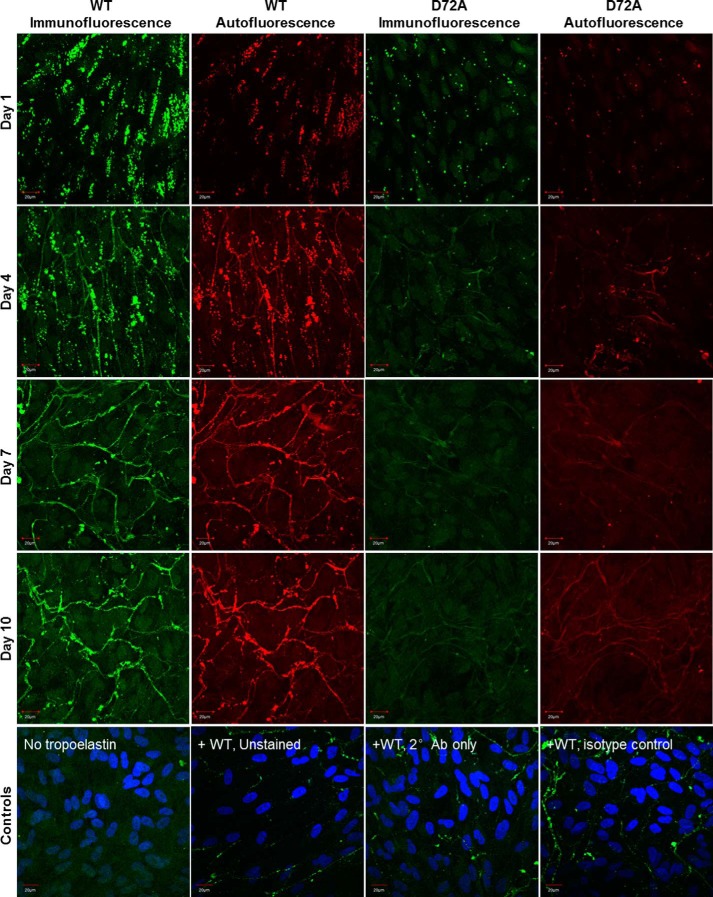
**Confocal microscope images of elastic fibers formed from exogenous WT and D72A tropoelastin in ARPE-19 cultures, at 1, 4, 7, and 10 days after tropoelastin addition.** Immunofluorescence and autofluorescence signals from each representative field of view are shown. Staining controls include cells with no added tropoelastin, and samples with added tropoelastin but are unstained, have no primary antibody, or have a nonspecific primary mouse antibody with the anti-mouse secondary antibody. Staining controls show immunofluorescence signals as well as DAPI-stained cell nuclei. *Scale bar,* 20 μm.

**FIGURE 6.**
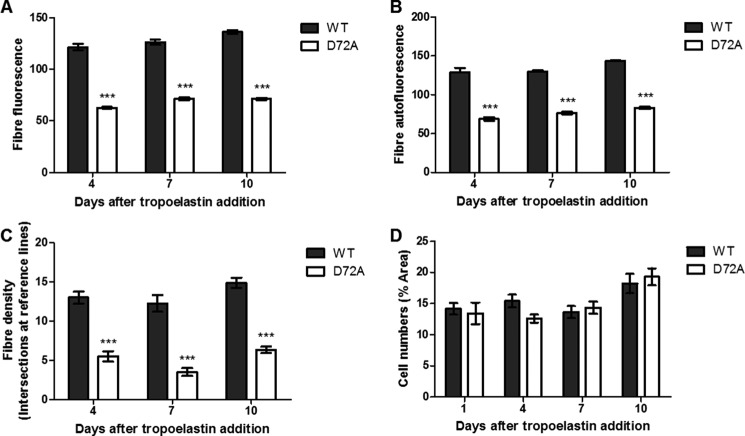
**Analysis of WT and D72A elastic fiber properties in ARPE-19 cells.** The immunofluorescence (*A*), autofluorescence (*B*), and abundance (*C*) of the assembled elastic fibers are indicated. *D,* comparable cell numbers, represented by cell area, in cultures with exogenous WT or D72A. ***, *p* < 0.001.

The elastogenic capability of WT and D72A tropoelastin was also assessed in ARPE-19 human retinal pigmented epithelial cells, a cell line that naturally expresses major extracellular matrix components but not tropoelastin (Fig. 5). One day after the addition of WT and D72A tropoelastin to the culture medium of these cells, WT spherules were likewise organized linearly, although the D72A spherules were scattered throughout the extracellular space. Four days after tropoelastin addition, an expansive WT elastic fiber network had assembled. Several D72A elastic fibers were also visible, but they were significantly fewer in number and exhibited significantly decreased immunofluorescence and autofluorescence than WT fibers (Fig. 6, *A–C*). The same pattern was maintained until 7 and 10 days after tropoelastin addition. D72A elastic fibers consistently stained 45–48% less intensely, displayed 41–47% reduced autofluorescence, and exhibited 57–71% decreased abundance compared with WT fibers.

In all cases, cell numbers were comparable between samples to which WT and D72A were added (Fig. 6*D*), suggesting that the differences in elastic fiber properties were not due to differences in cell density. In addition, control samples without exogenous tropoelastin showed no elastic fiber formation. Staining controls containing WT tropoelastin but with missing or nonspecific antibodies showed faintly visible elastic fibers due to elastin autofluorescence.

##### D72A Tropoelastin N-terminal Region Binds Differentially to a Domain 6-specific Antibody

**FIGURE 7.**
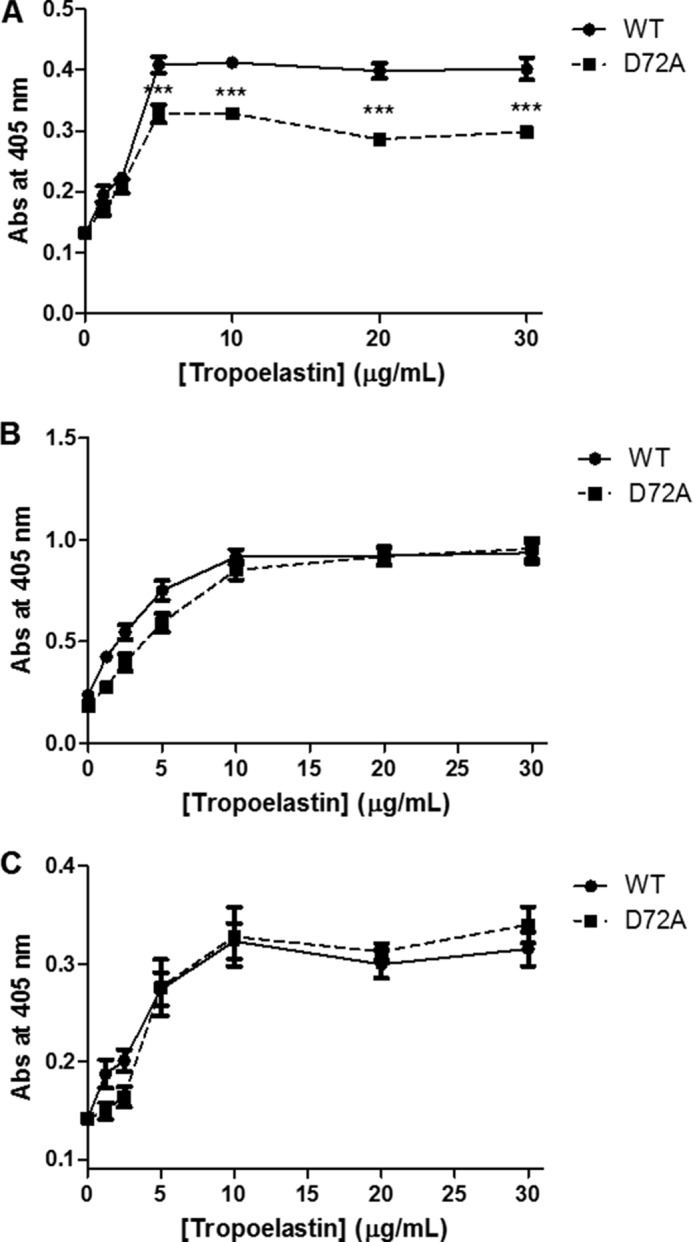
**Detection of surface-bound WT and D72A tropoelastin by antibodies targeted against domain 6 (*A*), domain 24 (*B*), and domain 36 (*C*).**

Antibodies targeted against different regions within the tropoelastin molecule were used to examine potential conformational changes between WT and D72A. Immunodetection of surface-coated tropoelastin increased with increasing tropoelastin concentrations until saturation at 5–10 μg/ml tropoelastin. Significantly reduced binding levels were detected for D72A compared with WT when using a specific antibody against domain 6, which encompasses the mutation site (Fig. 7*A*). No differences were observed between WT and D72A with antibodies targeted against the central domain 24 (Fig. 7*B*) or the C-terminal domain 36 (Fig. 7*C*).

##### D72A Tropoelastin Has Similar Secondary Structure Composition to WT

**FIGURE 8.**
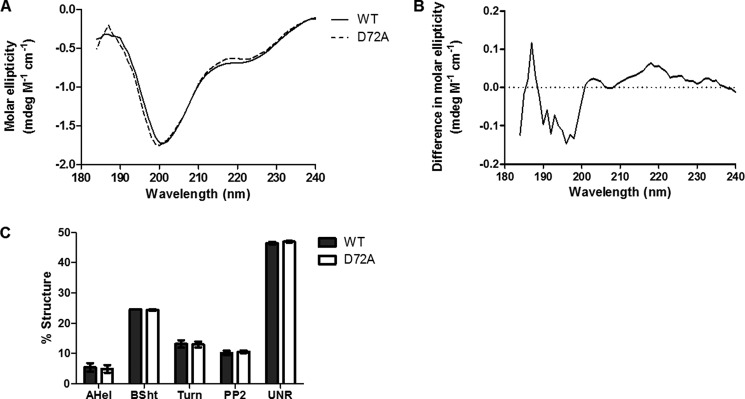
**Secondary structure analysis of WT and D72A tropoelastin.** *A,* far-UV CD spectra from 184 to 240 nm. *B,* difference obtained by subtracting the WT spectrum from the D72A spectrum. *C,* secondary structure composition of both species estimated by the CONTINLL and CDSSTR algorithms. The percentage of α-helix (*AHel*), β-sheet (*BSht*), β-turn, polyproline II helix (*PP2*), and unresolved structures are indicated.

The far-UV circular dichroism (CD) spectra of WT and D72A displayed similar features characteristic of tropoelastin, including a large negative peak at ∼200 nm and a negative shoulder at ∼220 nm, although there were slight differences in the scattering profile around these wavelengths (Fig. 8, *A* and *B*). The secondary structure compositions of WT and D72A tropoelastin were calculated to be similar, consisting of 5.4 and 5.0% α-helix, 24.6 and 24.4% β-sheet, 13.2 and 13.0% turn region, 10.2 and 10.6% polyproline II helix, and 46.5 and 47% unresolved structures, respectively (Fig. 8*C*).

##### D72A Tropoelastin Shows Altered N-terminal Region Conformation

**FIGURE 9.**
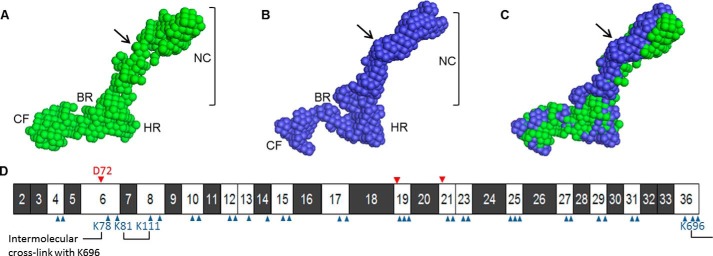
*A* and *B,* solution structures of WT (*green*) and D72A (*blue*) tropoelastin obtained by small angle x-ray scattering. The N-terminal coil (*NC*) region, hinge region (*HR*), bridge region (*BR*), and C-terminal foot (*CF*) region are indicated. *C,* aligned WT and D72A structures. *Arrows* indicate the bend/twist in the D72A N-terminal region resulting in an altered curvature compared with the native shape. *D,* domain structure of WT human tropoelastin showing hydrophobic (*black*) and hydrophilic (*white*) domains. The locations of all negatively charged (*red arrowhead*) and positively charged (*blue arrowhead*) residues are indicated. Lysine residues in domain 6 involved in intramolecular or intermolecular cross-linking are also identified.

The solution structures of the tropoelastin constructs were determined via small angle x-ray scattering. Both WT and D72A shapes possessed similar features comprising an elongated N-terminal coil segment that branches into a hinge region and a bridge region linked to the C-terminal foot region (Fig. 9, *A–C*). Alignment of the WT and D72A structures indicated similar bridge region lengths and, consequently, a comparable distance from the central axis to the C-terminal region of each molecule. However, the WT and D72A N-terminal regions displayed conformational differences. The mid-section of the D72A coil region displayed a more prominent bend or twist, resulting in an altered curvature compared with that of WT tropoelastin.

## DISCUSSION

Through the functional and structural characterization of a D72A human tropoelastin mutant, we sought to elucidate the significance of the N-terminal negatively charged Asp-72 residue, and that of the N-terminal coil region as a whole, in the assembly of tropoelastin into elastic fibers.

Coacervation represents the first crucial step of elastogenesis, and the ability of tropoelastin molecules to coacervate greatly impacts upon their assembly into elastic fibers. D72A exhibited temperature-dependent self-association similar to WT but had increased temperature and temporal requirements for this size transition. The coacervation ability of tropoelastin species conventionally correlates with protein hydrophobicity (31, 32). However, WT and D72A possess an equal number of hydrophobic regions, and the substitution of the charged aspartate in the mutant with a more hydrophobic alanine residue should maintain or improve coacervation efficiency. The decreased propensity of D72A for coacervation is therefore likely unrelated to altered protein hydropathy and more likely is due to structural differences between WT and D72A that alter the exposure or proximity of interacting hydrophobic domains.

Apart from the differences in the coacervation requirements of WT and D72A, the full-sized WT coacervate spherules were more than four times larger than those of D72A, even at high temperatures, which allow WT and D72A coacervation to proceed fully and at a similar rate. The smaller size of D72A assemblies implies a different self-organization of mutant constructs that precludes coacervation to the same extent as WT.

It is surprising that a single amino acid mutation in the hydrophilic domain 6 of tropoelastin impairs its ability to coacervate normally, because the process is thought to be dominated by the large hydrophobic domains in the central region of tropoelastin, such as domains 18, 26, 28, and 30 (5, 33). Furthermore, multiple site mutations in the more proximal domain 20 do not affect the coacervation profile of full-length tropoelastin variants (11), concordant with the relatively high allowance for sequence polymorphisms in the hydrophobic regions of tropoelastin (34). The impact of the D72A mutation on tropoelastin coacervation implicates the involvement of the N-terminal segment in this initial assembly process. This model is supported by the ability of elastin peptides containing domains 2–7 to coacervate into structures reminiscent of those formed by the full-length monomer (6).

The ability of tropoelastin to be cross-linked also correlates with its incorporation into insoluble elastic fibers (15). Although both WT and D72A can be chemically cross-linked, the resulting hydrogels differed markedly in morphological and functional properties. The open, porous network of the WT construct, which is consistent with the fibrous nature of physiological elastin (35), contrasts against the dense, layered structure of the D72A material. The reduced porosity of the D72A material reflects its significantly reduced water uptake compared with the WT. The distinct hydrogel properties strongly suggest differences in the cross-linking process undergone by WT and D72A monomers.

Tropoelastin cross-linking, whether enzymatic or chemical, is known to be enriched in the central region spanning domains 19–25 (8, 9). The abnormal cross-linking of D72A tropoelastin may result from atypical assembly of the initial coacervate species, as intermolecular misalignments can affect the formation of native cross-links. In addition, domain 6 has been shown to directly participate in cross-linking. Its Lys-78 residue is thought to bind to the Lys-696 of domain 36 in an intermolecular linkage (9), although its Lys-81 contacts the Lys-111 of domain 8 via an intramolecular bond (8). The involvement of domain 6 in cross-linking is consistent with the surface accessibility of the N-terminal region (36) and is supported by the presence of a protease-susceptible Lys-152 site in nearby domain 10 (37). The participation of domain 6 in cross-linking therefore suggests that the process can be affected by local conformational shifts arising from the D72A mutation that disturb the juxtaposition of interacting lysine residues. Depending on the scale of the structural changes, cross-link formation within the proximal domains 12–14 (8, 9) may also be altered.

WT and D72A tropoelastin equally promote the adhesion of human dermal fibroblasts, which bind via integrins to tropoelastin central domains 17 and 18 (38) or C-terminal domain 36 (12). The comparable levels of fibroblast adhesion to WT and D72A therefore indicate similar accessibility of their central and C-terminal regions. The results further suggest that any structural changes in D72A are most likely localized to the N-terminal segment, as conformational shifts within the bridge region also influence the orientation of the C terminus (1).

Exogenous tropoelastin added to the culture media of elastogenic cells can assemble into elastic fibers (39). WT developed into well defined elastic fibers within the extracellular matrix of human dermal fibroblasts and ARPE-19 cells. In contrast, D72A did not form elastic fibers with fibroblasts and formed only a few fibers with atypical properties with ARPE-19 cells. The decreased immunofluorescence of D72A fibers with ARPE-19 cells may arise from fewer tropoelastin molecules or reduced accessibility of antibody-targeted motifs, consistent with differential monomer packing within the elastic fiber. The decreased autofluorescence of D72A fibers also implicates altered elastin cross-linking (40). The decreased elastogenic capability of D72A is not due to impaired cellular binding proposed to be essential for the anchorage of the elastic network (12). Rather, it is a likely consequence of demonstrably impaired macromolecular assembly and potentially deficient interactions with extracellular matrix components. The N-terminal domains 4–6 of tropoelastin contain a high affinity binding site for the microfibrillar protein fibrillin-1 (7). Local structural changes in D72A occurring around domain 6 may therefore negatively affect tropoelastin deposition on the microfibrillar scaffold and subsequent cross-linking into stable elastic fibers.

The altered self-assembly and elastogenic properties of D72A provide strong support for the presence of conformational changes in the monomer. Antibodies targeted against specific tropoelastin regions were used as a preliminary tool for probing the location and extent of these potential structural changes. Compared with the WT species, D72A displayed equal levels of detection by both the BA4 and the anti-C-terminal antibodies. The BA4 anti-elastin antibody predominantly targets the VGVAPG hexapeptide in domain 24 and to a lesser extent other hydrophobic sequences throughout the molecule that follow the *X*G*XX*PG or *X*G*X*PG*X* motifs (41). The anti-C-terminal antibody specifically targets tropoelastin domain 36. Comparable binding of both antibodies to WT and D72A suggests preservation of the central and C-terminal region structures in the D72A mutant. In contrast, the significantly reduced binding of the anti-domain 6 antibody to D72A compared with WT strongly points to the likelihood of a conformational shift in this region of D72A.

The overall secondary structure compositions of WT and D72A were calculated to be similar, with a major percentage allocated to unordered regions consistent with the flexible nature of tropoelastin (42, 43) and decreasing amounts of β-sheet, β-turn, polyproline II helix, and α-helix structures. However, a direct comparison of the WT and D72A CD spectra may not resolve small local differences in secondary structure. Subtracting the WT spectrum from that of D72A may help magnify such differences. Using this approach, D72A shows, at most, a slight reduction in α-helical structure with a concurrent increase in unordered regions. The propensity for α-helix formation has been directly correlated to the number of consecutive alanine residues (44). The aspartate-to-alanine mutation in the D72A construct would adjoin the preceding Ala-71 residue to the downstream native Ala-73 to Ala-76 tetra-alanines to form a hexa-alanine sequence that would theoretically have a higher predisposition for α-helix formation. However, the stability of this nascent helix structure may be affected by the substitution of the N-terminal α-helical capping residue from the native aspartate at position 72 to a valine at position 70 in the mutant. Aspartate is the second most preferred N-capping residue in α-helical peptides, whereas valine is the third least favorable (45). Given the role of α-helices in positioning lysine residues for cross-linking (46), changes in the α-helical content of domain 6 may help explain the impaired D72A assembly.

The WT and D72A small angle x-ray scattering solution shapes showed the main structural features previously reported for human tropoelastin (47). Alignment of the WT and D72A models revealed a series of subtle conformational rearrangements in the N-terminal coil region that encompasses the Asp-72 residue and domain 6. The D72A mutation is clearly associated with an altered curvature and orientation of the segment spanning the N terminus to the mid-section of the coil region. A contributing factor to this structural deviation may be the destabilization of α-helices within domain 6. These results suggest for the first time a role for the Asp-72 residue in maintaining the native conformation of the tropoelastin N-terminal coil region, potentially by stabilizing local secondary structure and/or contacting proximal residues via charge interactions. The Asp-72 residue may interact with one of the two positively charged lysines in domain 8 to constrain this region and allow the formation of the reported intramolecular linkage between Lys-81 of domain 6 and Lys-111 of domain 8 (8) during *in vivo* cross-linking (Fig. 9*D*).

We propose a model where the N-terminal structural change at the monomer level accounts for the altered assembly of D72A tropoelastin. Protein hydration changes associated with the conformational shift can affect the thermodynamic requirements of coacervation, whereas modifications to the relative position of domains can decrease the efficiency of self-association and impede native molecular packing within the assembled species. In this model, the misalignment of cross-linking domains contributes to formation of aberrant intramolecular and intermolecular linkages, particularly those involving domain 6 and neighboring domains. Atypical cross-linking of the D72A species is consistent with the structural and functional abnormalities of its hydrogel constructs. Evidence for a domain 6-domain 36 cross-link (9) strengthens the head-to-tail model of tropoelastin assembly (47), which suggests that N-terminal conformational changes can drastically hinder monomer propagation into fiber structures as observed experimentally. The impaired elastogenic ability of D72A tropoelastin directly supports a model in which the Asp-72 residue critically maintains the structure of the tropoelastin N-terminal region for the normal assembly and architecture of elastic fibers.

In light of the substantial effects seen here, we would expect a marked elastin pathology associated with a mutation at the tropoelastin Asp-72 site. If such a mutation were viable, the tropoelastin variant would be expected to display impaired coacervation and aberrant cross-linking behavior that cumulatively results in deficient or atypical elastic fiber assembly. Abnormal elastogenesis is resonant of the cutis laxa pathology, which is typically associated with missense or frameshift mutations in the tropoelastin C-terminal region, but a more severe, recessive phenotype has recently also been correlated to a point mutation in the tropoelastin N-terminal domain 12 (11). Similar to the clinical consequences of cutis laxa-associated mutations, the features of tropoelastin with a nonconservative Asp-72 mutation would also be expected to result in a decreased ability of the elastic matrix to fulfill the functional requirements of the tissue environment.

## References

[1] Yeo G. C. Baldock C. Tuukkanen A. Roessle M. Dyksterhuis L. B. Wise S. G. Matthews J. Mithieux S. M. Weiss A. S. (2012) Tropoelastin bridge region positions the cell-interactive C terminus and contributes to elastic fiber assembly. Proc. Natl. Acad. Sci. U.S.A. 109, 2878–2883 22328151 10.1073/pnas.1111615108 PMC3286909

[2] Dyksterhuis L. B. Baldock C. Lammie D. Wess T. J. Weiss A. S. (2007) Domains 17–27 of tropoelastin contain key regions of contact for coacervation and contain an unusual turn-containing crosslinking domain. Matrix Biol. 26, 125–135 17129717 10.1016/j.matbio.2006.10.002

[3] Brown-Augsburger P. Broekelmann T. Rosenbloom J. Mecham R. P. (1996) Functional domains on elastin and microfibril-associated glycoprotein involved in elastic fibre assembly. Biochem. J. 318, 149–155 8761465 10.1042/bj3180149 PMC1217601

[4] Kozel B. A. Wachi H. Davis E. C. Mecham R. P. (2003) Domains in tropoelastin that mediate elastin deposition *in vitro* and *in vivo*. J. Biol. Chem. 278, 18491–18498 12626514 10.1074/jbc.M212715200

[5] Jensen S. A. Vrhovski B. Weiss A. S. (2000) Domain 26 of tropoelastin plays a dominant role in association by coacervation. J. Biol. Chem. 275, 28449–28454 10862774 10.1074/jbc.M004265200

[6] Ostuni A. Bochicchio B. Armentano M. F. Bisaccia F. Tamburro A. M. (2007) Molecular and supramolecular structural studies on human tropoelastin sequences. Biophys. J. 93, 3640–3651 17693470 10.1529/biophysj.107.110809 PMC2072060

[7] Clarke A. W. Wise S. G. Cain S. A. Kielty C. M. Weiss A. S. (2005) Coacervation is promoted by molecular interactions between the PF2 segment of fibrillin-1 and the domain 4 region of tropoelastin. Biochemistry 44, 10271–10281 16042404 10.1021/bi050530d

[8] Mithieux S. M. Wise S. G. Raftery M. J. Starcher B. Weiss A. S. (2005) A model two-component system for studying the architecture of elastin assembly *in vitro*. J. Struct. Biol. 149, 282–289 15721582 10.1016/j.jsb.2004.11.005

[9] Wise S. G. Mithieux S. M. Raftery M. J. Weiss A. S. (2005) Specificity in the coacervation of tropoelastin: solvent exposed lysines. J. Struct. Biol. 149, 273–281 15721581 10.1016/j.jsb.2004.11.006

[10] Brown-Augsburger P. Tisdale C. Broekelmann T. Sloan C. Mecham R. P. (1995) Identification of an elastin cross-linking domain that joins three peptide chains. Possible role in nucleated assembly. J. Biol. Chem. 270, 17778–17783 7629078 10.1074/jbc.270.30.17778

[11] Mégarbané H. Florence J. Sass J. O. Schwonbeck S. Foglio M. de Cid R. Cure S. Saker S. Mégarbané A. Fischer J. (2009) An autosomal-recessive form of cutis laxa is due to homozygous elastin mutations, and the phenotype may be modified by a heterozygous fibulin 5 polymorphism. J. Invest. Dermatol. 129, 1650–1655 19194475 10.1038/jid.2008.450

[12] Bax D. V. Rodgers U. R. Bilek M. M. Weiss A. S. (2009) Cell adhesion to tropoelastin is mediated via the C-terminal GRKRK motif and integrin αVβ3. J. Biol. Chem. 284, 28616–28623 19617625 10.1074/jbc.M109.017525 PMC2781405

[13] Akhtar K. Broekelmann T. J. Song H. Turk J. Brett T. J. Mecham R. P. Adair-Kirk T. L. (2011) Oxidative modifications of the C-terminal domain of tropoelastin prevent cell binding. J. Biol. Chem. 286, 13574–13582 21321118 10.1074/jbc.M110.192088 PMC3075703

[14] Broekelmann T. J. Kozel B. A. Ishibashi H. Werneck C. C. Keeley F. W. Zhang L. Mecham R. P. (2005) Tropoelastin interacts with cell-surface glycosaminoglycans via its COOH-terminal domain. J. Biol. Chem. 280, 40939–40947 16192266 10.1074/jbc.M507309200

[15] Sato F. Wachi H. Ishida M. Nonaka R. Onoue S. Urban Z. Starcher B. C. Seyama Y. (2007) Distinct steps of cross-linking, self-association, and maturation of tropoelastin are necessary for elastic fiber formation. J. Mol. Biol. 369, 841–851 17459412 10.1016/j.jmb.2007.03.060

[16] Bedell-Hogan D. Trackman P. Abrams W. Rosenbloom J. Kagan H. (1993) Oxidation, cross-linking, and insolubilization of recombinant tropoelastin by purified lysyl oxidase. J. Biol. Chem. 268, 10345–10350 8098038

[17] Piontkivska H. Zhang Y. Green E. D., NISC Comparative Sequencing Program, and Elnitski L. (2004) Multi-species sequence comparison reveals dynamic evolution of the elastin gene that has involved purifying selection and lineage-specific insertions/deletions. BMC Genomics 5, 31 15149554 10.1186/1471-2164-5-31 PMC436053

[18] Wu W. J. Weiss A. S. (1999) Deficient coacervation of two forms of human tropoelastin associated with supravalvular aortic stenosis. Eur. J. Biochem. 266, 308–314 10542079 10.1046/j.1432-1327.1999.00891.x

[19] Long J. L. Tranquillo R. T. (2003) Elastic fiber production in cardiovascular tissue-equivalents. Matrix Biol. 22, 339–350 12935818 10.1016/s0945-053x(03)00052-0

[20] Taddese S. Weiss A. S. Neubert R. H. Schmelzer C. E. (2008) Mapping of macrophage elastase cleavage sites in insoluble human skin elastin. Matrix Biol. 27, 420–428 18334288 10.1016/j.matbio.2008.02.001

[21] Schmelzer C. E. Getie M. Neubert R. H. (2005) Mass spectrometric characterization of human skin elastin peptides produced by proteolytic digestion with pepsin and thermitase. J. Chromatogr. A 1083, 120–126 16078697 10.1016/j.chroma.2005.06.034

[22] Getie M. Schmelzer C. E. Neubert R. H. (2005) Characterization of peptides resulting from digestion of human skin elastin with elastase. Proteins 61, 649–657 16161116 10.1002/prot.20643

[23] Uitto J. (1979) Biochemistry of the elastic fibers in normal connective tissues and its alterations in diseases. J. Invest. Dermatol. 72, 1–10 368254 10.1111/1523-1747.ep12530093

[24] Urry D. W. Sugano H. Prasad K. U. Long M. M. Bhatnagar R. S. (1979) Prolyl hydroxylation of the polypentapeptide model of elastin impairs fiber formation. Biochem. Biophys. Res. Commun. 90, 194–198 496971 10.1016/0006-291x(79)91608-5

[25] Debelle L. Tamburro A. M. (1999) Elastin: molecular description and function. Int. J. Biochem. Cell Biol. 31, 261–272 10216959 10.1016/s1357-2725(98)00098-3

[26] Vrhovski B. Jensen S. Weiss A. S. (1997) Coacervation characteristics of recombinant human tropoelastin. Eur. J. Biochem. 250, 92–98 9431995 10.1111/j.1432-1033.1997.00092.x

[27] Sreerama N. Woody R. W. (2000) Estimation of protein secondary structure from circular dichroism spectra: Comparison of CONTIN, SELCON, and CDSSTR methods with an expanded reference set. Anal. Biochem. 287, 252–260 11112271 10.1006/abio.2000.4880

[28] Konarev P. V. Volkov V. V. Sokolova A. V. Koch M. H. Svergun D. (2003) PRIMUS–a Windows-PC based system for small-angle scattering data analysis. J. Appl. Crystallogr. 36, 1277–1282

[29] Svergun D. I. Petoukhov M. V. Koch M. H. (2001) Determination of domain structure of proteins from x-ray solution scattering. Biophys. J. 80, 2946–2953 11371467 10.1016/S0006-3495(01)76260-1 PMC1301478

[30] Volkov V. V. Svergun D. I. (2003) Uniqueness of ab-initio shape determination in small-angle scattering. J. Appl. Crystallogr. 36, 860–864 10.1107/S0021889809000338 PMC5023043 27630371

[31] Miao M. Bellingham C. M. Stahl R. J. Sitarz E. E. Lane C. J. Keeley F. W. (2003) Sequence and structure determinants for the self-aggregation of recombinant polypeptides modeled after human elastin. J. Biol. Chem. 278, 48553–48562 14500713 10.1074/jbc.M308465200

[32] Urry D. W. (2004) The change in Gibbs free energy for hydrophobic association–derivation and evaluation by means of inverse temperature transitions. Chem. Phys. Lett. 399, 177–183

[33] Toonkool P. Jensen S. A. Maxwell A. L. Weiss A. S. (2001) Hydrophobic domains of human tropoelastin interact in a context-dependent manner. J. Biol. Chem. 276, 44575–44580 11564742 10.1074/jbc.M107920200

[34] Bashir M. M. Indik Z. Yeh H. Ornstein-Goldstein N. Rosenbloom J. C. Abrams W. Fazio M. Uitto J. Rosenbloom J. (1989) Characterization of the complete human elastin gene. J. Biol. Chem. 264, 8887–8891 2722804

[35] Pasquali-Ronchetti I. Baccarani-Contri M. (1997) Elastic fiber during development and aging. Microsc. Res. Tech. 38, 428–435 9297692 10.1002/(SICI)1097-0029(19970815)38:4<428::AID-JEMT10>3.0.CO;2-L

[36] Heinz A. Jung M. C. Duca L. Sippl W. Taddese S. Ihling C. Rusciani A. Jahreis G. Weiss A. S. Neubert R. H. Schmelzer C. E. (2010) Degradation of tropoelastin by matrix metalloproteinases-cleavage site specificities and release of matrikines. FEBS J. 277, 1939–1956 20345904 10.1111/j.1742-4658.2010.07616.x

[37] Waterhouse A. Bax D. V. Wise S. G. Yin Y. Dunn L. L. Yeo G. C. Ng M. K. Bilek M. M. Weiss A. S. (2011) Stability of a therapeutic layer of immobilized recombinant human tropoelastin on a plasma-activated coated surface. Pharm. Res. 28, 1415–1421 21103913 10.1007/s11095-010-0327-z

[38] Lee P. Bax D. V. Bilek M. M. Weiss A. S. (2014) A novel cell adhesion region in tropoelastin that mediates attachment to integrin αVβ5. J. Biol. Chem. 289, 1467–1477 24293364 10.1074/jbc.M113.518381 PMC3894329

[39] Stone P. J. Morris S. M. Griffin S. Mithieux S. Weiss A. S. (2001) Building elastin–incorporation of recombinant human tropoelastin into extracellular matrices using nonelastogenic Rat-1 fibroblasts as a source for lysyl oxidase. Am. J. Respir. Cell Mol. Biol. 24, 733–739 11415939 10.1165/ajrcmb.24.6.4304

[40] Wang H. W. Simianu V. Locker M. J. Sturek M. Cheng J. X. (2008) Imaging arterial cells, atherosclerosis, and restenosis by multimodal nonlinear optical microscopy. Proc. SPIE 6860, 68600W; 10.1117/12.763604

[41] Grosso L. E. Scott M. (1993) Peptide sequences selected by BA4, a tropoelastin-specific monoclonal antibody, are ligands for the 67-kilodalton bovine elastin receptor. Biochemistry 32, 13369–13374 8241194 10.1021/bi00211a052

[42] Dyksterhuis L. B. Carter E. A. Mithieux S. M. Weiss A. S. (2009) Tropoelastin as a thermodynamically unfolded premolten globule protein: the effect of trimethylamine *N*-oxide on structure and coacervation. Arch. Biochem. Biophys. 487, 79–84 19501564 10.1016/j.abb.2009.06.001

[43] Muiznieks L. D. Weiss A. S. (2007) Flexibility in the solution structure of human tropoelastin. Biochemistry 46, 8196–8205 17567153 10.1021/bi700139k

[44] Tamburro A. M. Pepe A. Bochicchio B. (2006) Localizing α-helices in human tropoelastin: assembly of the elastin “puzzle.” Biochemistry 45, 9518–9530 16878986 10.1021/bi060289i

[45] Doig A. J. Baldwin R. L. (1995) N- and C-capping preferences for all 20 amino acids in α-helical peptides. Protein Sci. 4, 1325–1336 7670375 10.1002/pro.5560040708 PMC2143170

[46] Bochicchio B. Pepe A. (2011) Role of polyproline II conformation in human tropoelastin structure. Chirality 23, 694–702 22135799 10.1002/chir.20979

[47] Baldock C. Oberhauser A. F. Ma L. Lammie D. Siegler V. Mithieux S. M. Tu Y. Chow J. Y. Suleman F. Malfois M. Rogers S. Guo L. Irving T. C. Wess T. J. Weiss A. S. (2011) Shape of tropoelastin, the highly extensible protein that controls human tissue elasticity. Proc. Natl. Acad. Sci. U.S.A. 108, 4322–4327 21368178 10.1073/pnas.1014280108 PMC3060269

